# Novel imaging technologies for characterization of microbial extracellular polysaccharides

**DOI:** 10.3389/fmicb.2015.00525

**Published:** 2015-05-28

**Authors:** Magnus B. Lilledahl, Bjørn T. Stokke

**Affiliations:** Biophysics and Medical Technology, Department of Physics, The Norwegian University of Science and TechnologyTrondheim, Norway

**Keywords:** AFM, high resolution AFM, superresolution optical microscopy, SHG

## Abstract

Understanding of biology is underpinned by the ability to observe structures at various length scales. This is so in a historical context and is also valid today. Evolution of novel insight often emerges from technological advancement. Recent developments in imaging technologies that is relevant for characterization of extraceullar microbiological polysaccharides are summarized. Emphasis is on scanning probe and optical based techniques since these tools offers imaging capabilities under aqueous conditions more closely resembling the physiological state than other ultramicroscopy imaging techniques. Following the demonstration of the scanning probe microscopy principle, novel operation modes to increase data capture speed toward video rate, exploitation of several cantilever frequencies, and advancement of utilization of specimen mechanical properties as contrast, also including their mode of operation in liquid, have been developed on this platform. Combined with steps in advancing light microscopy with resolution beyond the far field diffraction limit, non-linear methods, and combinations of the various imaging modalities, the potential ultramicroscopy toolbox available for characterization of exopolysaccharides (EPS) are richer than ever. Examples of application of such ultramicroscopy strategies range from imaging of isolated microbial polysaccharides, structures being observed when they are involved in polyelectrolyte complexes, aspects of their enzymatic degradation, and cell surface localization of secreted polysaccharides. These, and other examples, illustrate that the advancement in imaging technologies relevant for EPS characterization supports characterization of structural aspects.

## Introduction

Polymers are abundant constituents of microbes and host a variety of specific functions. Among these, extracellular polysaccharides, or exopolysaccharides (EPS), constitute a group of carbohydrate based polymers, secreted from the bacteria that represent an interface to the environment 1. Exopolysaccharrides are generally thought to be released from the bacterial surface at variance with surface bound polysaccharides. Various types of microorganisms produce exopolysaccarides. These organisms exist in nature as environmental microbes and/or pathogens for humans. Additionally, they are also utilized industrially where their successful biotechnological fermentation yields efficient production of large quantitities of EPS. Bacterial exopolysccharides can be grouped into homo- and heteropolysaccharides. Cellulose is one example of a homopolysaccharide. Xanthan, gellan gum, succinoglycan, and alginates are examples of heteropolysaccharides. EPS posess various functionalities in their natural habitat, whereas fermented microbial EPS are applied in a wide range of application, where the functionality are indeed underpinned by their structure, but the exploited function is not necessarily coinciding with their original functionality. Application of imaging strategies to EPS is directed toward elucidation of their structure at various length scales and determination of aspects of functionalproperties.

The paper is organized as outlined in the following, we summarize briefly imaging modalities developed based on scanning probe platforms, mainly the atomic force microcsopy (AFM), their operation modes and associated contrast mechanisms. Recent developments of the AFM is imaging platform include novel operation modes to increasing data capture speed toward video rate, exploitation of several cantilever frequencies, and advancement of using specimen mechanical properties as contrast. In addition, the discovery of tip-enhanced Raman spectroscopy (TERS) bolster the development of probe based scanning technologies utilizing molecular vibration signals from nanoscale domains of the specimens. This review then summarizes advancement in optical microscopy with resolution beyond the far field diffraction limit, optical non-linear methods, and combinations of the various imaging modalities. The overview illustrate applications of these imaging modalities with reference to examples of results either reported for EPS or being relevant for future applications within the EPS field. Issues related to preparation of the specimens are briefly presented within the examples. The capability of AFM and optical based techniques to aquire high resolution information on specimens under hydrated conditions is different from the sample preparation requirements for electron microscopy (e.g., Vu et al., 2009, related to preparation of EPS). The selection of focus in this review on AFM and optically based imaging modalities is due to the combination of novel developments in these field and their capabilities to realize high resolution imaging also under aqueous conditions.

## Scanning Probe Based Microscopy and their Applications to Microbial Extracellular Polysaccharides

The imaging principle of the atomic force microscopy (AFM; Binnig et al., 1986) is based on raster scanning of a sharp tip at the end of a small cantilever with sub-nanometer resolution of the position control (Ellis et al., 2006). The deflection of the cantilever as perturbed by the localized interaction of the specimen and the tip is recorded and used as basis for the image generation. There are various operating principles of the AFM, where the most common ones are contact, intermittent contact, and non-contact mode. In the contact mode, the tip is scanned over the sample surface using a piezoelectrical scanner for positioning control and the same time the localized induced deflection of the cantilever is recorded, most commonly by monitoring the laser position of a reflected laser beam with a quadrant photodiode. Contact mode can in principle be operated in constant height or constant force, the latter invoking the feedback loop to maintain a constant net force at a user set value. Possible adverse effect associated with image acquisition induced sample relocation and deformation, in particular for soft samples like extracellular polysaccharides, has led to development of alternative imaging modes. The AFM tip is put into forced oscillations in both non-contact and intermittent contact mode. In the non-contact mode, the AFM tip is oscillated sufficiently close to the sample surface that mainly attractive sample-AFM tip interactions, but not steric repulsive interactions, affect the characteristic oscillation parameters of the cantilever. In the intermittent contact mode, the oscillating AFM tip is in contact with the sample surface at a certain fraction of the oscillation cycle (e.g., 5–15%) and thereby affects the oscillation parameters in a characteristic way. The feedback system is for most operations also engaged in the oscillatory mode, using, e.g., changes in oscillation amplitude as the feedback signal. Thus, topographs representing various features of the sample are obtained. For instance, the topographs obtained for the contact mode operation using the deflection of the cantilever as the feedback signal, can be understood as height isocontours representing the same force due to the AFM tip – sample interactions. There are analogous interpretations of the AFM topographs for the oscillatory modes. Irrespective of the scanning operation mode being contact or oscillation mode, the interpretation of EPS structures observed by AFM appears mainly to focus on the structures seen as polymer chain trajectories or overall conformation if the chain trajectory is not resolved.

**FIGURE 1:**
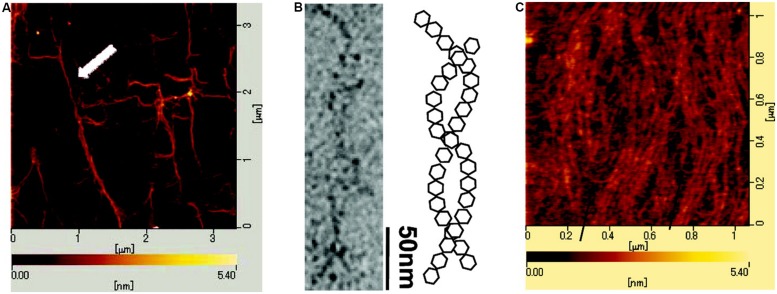
**Atomic force **(A,C)** and electron microscopic **(B)** images of cyanobacterial polysacaccharide sacran**. The atomic force microcsopy (AFM) topograph **(A)** was obtained from a 1 ppm sacran aqueous aliquot dried on mica. The white arrow highlights a bundle of sacran chains. **(B)** Transmisssion electron micrograph of sacran dried on carbon-coated Cu-grid and schematic illustration of intertwined (helical) structure adopted by sacran chains as a possible interpretation of the TEM micrograph. **(C)** AFM topograph of an aliquot of 0.1 mg/ml sacran aqueous solution dried on mica. Scale bars for lateral dimensions **(A–C)** and height **(A,C)** are indicated on the topographs. Reprinted with permission from Okajima et al. (2009). Copyright (2009) American Chemical Society.

One recent example of application of AFM imaging to EPS is the AFM topographs reported for sacran (Okajima et al., 2009). Sacran is a high molecular weight extracellular polysaccharide of the cyanobacteria *Aphanothece sacrum*. It is a heteropolysaccharide reported to consist of various monosaccharides Glc, Gal, Man, Xyl, Rha, Fuc, GalA, and GlcA and trace amounts of additional ones. Sacrans dominating anionic character is originating from 17 mol% of the sugar residues bearing carboxylate and 12 mol% bearing sulphate groups (Mitsumata et al., 2013). Samples of sacran were characterized by AFM to obtain information related to the physical structure of the polysaccharide chains. The AFM topograph of sacran dried from a 1 μg/ml (1 ppm) aqueous solution (**Figure 1A**) reveal rather elongated and apparantly interconnected structures, and for some regions also bundle-like appearance. The structural indication of an intertwined chain organization was further investigated using transmission electron microscopy, providing pictorial evidence that was interpreted in terms a two-chain bundle similar to a coil-coil structures well known for certain proteins. The possible variable pitch was observed to be in the range 20–120 nm. Increasing the sacran concentration in the aqueous solution to 0.1 mg/ml, drying an aliquot and imaging, yields an appearance with tendency for alignment of the chains (**Figure 1C**), but apperantly not increasing extent of bundling by further lateral associations as compared to the specimen dried from the less concentrated case.

Provided the polysaccharide chain trajectories are resolved in the topographs as obtained by AFM, one can extract quantitative information of chain stiffness by applying a statistical model. The persistence length, *L_p_*, of the polymer is a parameter reflecting the chain stiffness, and can be extracted from observed chain trajectories based on changes in tangent direction φ, as a function of the segment length *l.* Under the assumption that the chain is equilibrated in proximity of imaging surface and 2-dimensional chain statistics can be applied, the mean of the square of *φ* increases with the segment length in a way that allow determination of the persistence length (Frontali et al., 1979):

(1) $$\langle \varphi^{2}(l)\rangle =l/L_{p}$$

or alternatively:

(2) $$\langle \cos\left(\varphi (l)\right)\rangle =e^{-l/2L_{p}}$$

The mean (as depicted by the angle brackets) here imply accumulation of sufficient basis in terms of independent observations of φ at the various *l* for the statistics to be fulfilled, and equations supporting such a test have been provided (Frontali et al., 1979; Stokke and Brant, 1990). The model assumes homogeneous stiffness along the chain. To discern whether the observed trajectories correspond to the 2-dimensional chain statistics or not is a demanding task. In general, surfaces that interact with the macromolecules with different strengths have shown to affect the overall extension (Lamour et al., 2014), thus providing a practical handle to this issue. Data from the application of such a procedure have been reported for the bacterial polysaccharides xanthan (Camesano and Wilkinson, 2001) and succinoglycan (Balnois et al., 2000), which also include studies at different salt concentrations. Application of such a procedure appears to be difficult to the AFM topographs of sacran (**Figure 1**) due to the not clearly resolved chain trajectories. We have previously reviewed ultramicroscopy of polysaccharides using this approach (Stokke and Elgsaeter, 1994). There are a number of issues of the approach briefly described here to extract conformational parameters in a quantitative way based on observed trajectories. We refer the interested reader to the recent review (Gallyamov, 2011) for a more thorough discussion on the various assumptions and limitation of this approach.

Additional examples of application of AFM to interrogate EPS physical structure include effect of fermentation medium composition on the structure of EPS produced by *Lactobacillus rhamnosus* (Polak-Berecka et al., 2015), and effects of solvent and derivatization on the higher order structures of curdlan (Jin et al., 2006a,b). Cholesterol modified pullulan, the unmodified polymer being produced by the fungus *Aureobasidum pullulans* (Zalar et al., 2008) have a globular appearance in the AFM (Lee and Akiyoshi, 2004). The latter represent an example where the imaging technology was not able to provide direct information of the chain trajectory, nevertheless, the globular appearance is valuable qualitiative information in interpretation of other physical observables.

**FIGURE 2:**
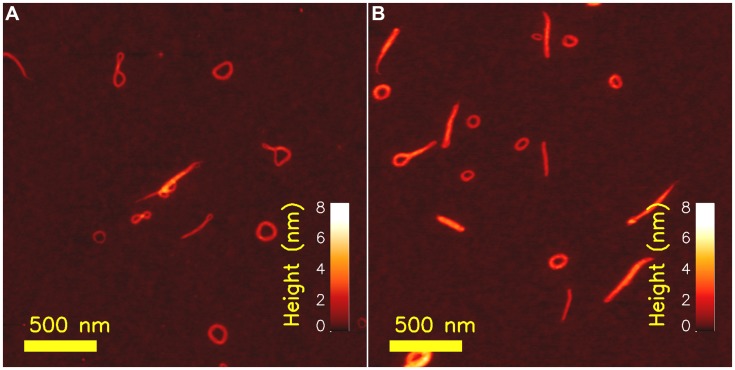
**Atomic force microcsopy topographs of chitosan– xanthan polyelectrolyte complexes (PECs) as prepared at low concentrations (xanthan concentration of 2 μg/ml and chitosan with degree of acetylation 0.49 at a concentration of 10 μg/ml) at room temperature and treated at 44%#x000B0;C for 30 min **(A)** and following annealing for 30 min at a temperature of 90%#x000B0;C (B)**. Reproduced with with permission from Maurstad and Stokke (2004). Copyright (2004) Wiley.

Atomic force microscopy applied to determine overall structure of polyelectrolyte complexes (PECs) of xanthan, an anionic, semiflexible extracellular polysaccharide, have contributed to the understanding of chain stiffness of PEC morphologies and their possible folding path toward a stable state. Xanthan is an EPS from *Xanthomonas sp*., and biotechnological production of xanthan by fermentation of *Xanthomonas campestris*, is one of the success stories of industrial scale EPS production. The primary structure of xanthan is a pentasaccharide repeating unit with two 1,4 linked β-D-Glcp residues making up cellulosic polymer backbone while β-D-Man, β-D-Glu, β-D-Man trisaccharides are linked to every second backbone residue (Jansson et al., 1975; Melton et al., 1975) thus making it appear with a comblike brushed architecture. Historically, there has been much controversy related to the molecular detail of the secondary structure of this polysaccharide, while there today appears to be consensus toward a duplex structure in the chiroptically detected ordered state prevailing at high ionic strength and low temperature (Stokke et al., 1998; Matsuda et al., 2009). In this ordered state, the xanthan behaves as a semiflexible polymer with persistence length of about 120 nm. Xanthan–chitosan PECs prepared at low concentration (1–2 μg/ml), prepared for AFM imaging on freshly cleaved mica and imaged by tapping mode AFM revealed various types of structures (**Figure 2**; Maurstad et al., 2003; Maurstad and Stokke, 2004). For complexes prepared using high molecular weight xanthan, toroidal species were present, although not all complexes were conforming to this well known structure observed for condensed DNA (Martin et al., 2000; Hud and Downing, 2001; Hud and Vilfan, 2005). The xanthan–chitosan AFM topographs were subjected to an image analysis process to quantitate fraction of species in dominating morphologies such as toroids, topologically straight (i.e., including also curved ones) and globular appearance. Thus, the asphericity index A (Eq. 3) was calculated for each of the identified PECs (Zifferer and Olaj, 1994; Zifferer, 1998; Maurstad et al., 2003):

(3) $$A=\frac{{\left(\lambda_{1}^{2}{\mathrm{-\lambda}}_{2}^{2}\right)}^{2}+{\left(\lambda_{2}^{2}{\mathrm{-\lambda}}_{3}^{2}\right)}^{2}+{\left(\lambda_{1}^{2}{\mathrm{-\lambda}}_{3}^{2}\right)}^{2}}{2{\left(\lambda_{1}^{2}{\mathrm{+\lambda}}_{2}^{2}{\mathrm{+\lambda}}_{3}^{2}\right)}^{2}}$$

where ëi, i = 1,3, are the eigenvalues of the tensor moment of inertia:

(4) $$X_{\mathrm{\alpha ,\beta}}=\frac{1}{N}\sum_{i=1}^{N}S_{i}^{\alpha }S_{i}^{\beta };\;\vec{S_{i}}=\vec{r_{i}}-{\vec{R}}_{CM};\mathrm{\alpha ,\beta}=x,y,z$$

Parameters $\overset{\to }{r_{i}}$ and ${\overset{\to }{R}}_{CM}$ in Eq. 4 depicts the positional co-ordinate of the i’th pixel element and the center of mass of the PEC, respectively. Parameter *A* is reported to adopt values of 0, 0.25, and 1.0 for the idealized sphere, an infinitely thin circle and rigid rod, respectively (Noguchi and Yoshikawa, 1998), and thus serve as a morphological sensitive parameter. Distributions of *A* extracted from a large number of xanthan–chitosan complexes prepared from a high molecular weight xanthan revealed a peak centered at *A* = 0.25 reflecting the toroidal like structure, and a tail in the distribution of A’s toward 1 reflecting rod-like PECs with different tortuosity (Maurstad et al., 2003; Maurstad and Stokke, 2004). Reduction of the molecular weight of xanthan below that needed for a toroidal morphology resulted in rod-like PECs with a concomitant suppression of the peak in the distribution of *A* around 0.25, and increase in the fraction of species with *A* closer to 1. Thus, the inclusion estimates of the asphericity indeces A provide quantitative characterization of distribution of species that (co-)exist in topographs.

Additionally, AFM imaging of heat-treated complexes formed between xanthan and chitosan provided indirect evidence of the folding pathway toward the toroid. Toward this end, the AFM topographs were considered as snap-shots of a particular state on its path toward a more stable state, stimulated either by increasing the temperature during a given duration of thermal treatment, or increasing duration at a given, elevated temperature. The fraction of species consisting of more than one loop, and often dangling ends, were observed to decrease, while the fraction of toroids was found to increase. Thus, the torus was suggested to represent the energetically favorable structure and that other morphologies observed at room temperature were metastable states driven toward the more stable state of a torus by the increased temperature. These observations have more recently been put in context with a numerical account of the collapse pathway occurring in polymers in poor solvents (Lappala and Terentjev, 2013).

While the above examples provide important information as deduced from the static observation of various chain trajectories arrested at various conformation during the immobilization process (preparation for AFM imaging), there is a growing interest in dynamics within EPS. Historically, AFM has been inherently slow in image acquisition, with capture time for individual frames in the order of minutes or larger. Although this depends on the scan size, these inherent slow frame rates limit the access to information on dynamics related to EPS. Progress in increasing the frame rates of AFM imaging is based on detailed insight, optimization, and re-engineering of key components of the AFMs, like smaller moving mass, reducing the size of the cantilever to increase resonance frequency, and improved feedback circuit. The technicalities of these AFM hardware issues, despite its vital importance for successful higher frame rates capture, is considered beyond the topic of this overview and we refer the interested reader to relevant literature on these topics (Ando et al., 2001, 2008; Ando, 2012).

**FIGURE 3:**
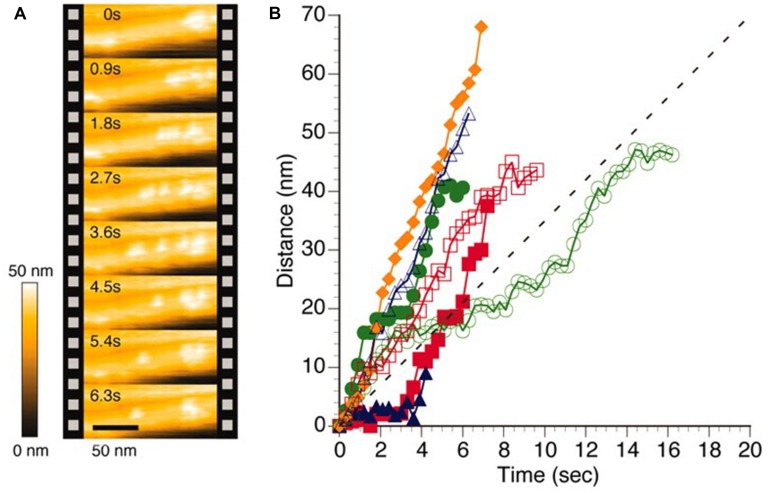
**High speed AFM imaging of *Trocoderma reesei* Cel7A cellulase acting on crystalline I_α_ cellulose. (A)** AFM topographs of the same area of the cellulose crystal imaged at intervals of 0.9 s revealing different localization of individual cellulases. **(B)** Time dependence from the initial position of individual cellulase positions when acting on cellulose crystal as deduced by image processing of HS-AFM topographs. The colored symbols reflect data extracted from individual enzymes whereas the dotted line depict an average velocity as reported (Igarashi et al., 2009). Reprinted with permission from Igarashi et al. (2011). Copyright (2011) AAAS.

One intriguing example of the application of high speed AFM to EPS is the characterization of cellulase induced degradation of crystalline cellulose specimen (Igarashi et al., 2011). Although the employed cellulose *I*_α_ crystal was obtained from processing of a sample from green alga *Cladohora* sp. is not a truly microbial EPS, the similarity of the specimen with bacterial cellulose is considered sufficiently close to include this example here. This cellulose crystal specimen was incubated on a graphite sample disk, and following addition of cellulase Cel7A from *Trichoderma reesei*, in buffered solution, sequential AFM imaging was performed at frame rates of 1–4 Hz. The movement of the Cel7A was observed only on the top of the crystalline cellulose specimen. Example data from their seminal report (**Figure 3**) shows relocation of individual cellobiohydrolase enzymes unidirectionally along the crystalline specimen, with a migration speed that is uneven and differs between the individual enzymes, but spread around the mean value as estimated from other average data. The data also revealed that individual enzymes does not exhibit a constant migration velocity, some enzymes were migrating without stopping within the observation areas, whereas others were apparently halting, suggested to arise from the Cel7A molecules exhibiting intermittently halt and go movements. The results were also discussed in terms of Cel7A interacting with the cellulose crystal in a productive and non-productive adsorption mode. Furthermore, detailed analysis of the dynamics revealed congestion between individual cellulases when moving on the crystalline specimen. As part of this, the inability of a particular enzyme to circumvent a stalled enzyme on the crystalline specimen was identified. Detailed molecular studies using high speed AFM imaging, such as that highlighted above, provide additional microscopic details on the statistical nature and congestion effects on the bioconversion of cellulose crystals that are not easily captured by other approaches. Application of HS-AFM in biological systems has recently been reviewed (Ando et al., 2014).

Direct imaging of the enzymatic polymerization of hyaluronan using an *in vitro* approach was recently reported (Mori et al., 2012). A sample of hyaluronic acid synthase isolate from *Pasteurella multocida* (pmHAS) was physiosorbed to mica or immobilized on an anionic lipid bilayer spread on mica. The immobilized pmHAS sample was imaged at various intervals when incubated with both UDP-GlcA and UDP-GlcNAc monomers, being the UDP forms of the monomers of the repeating structure of the hyaluronan structure. The spatiotemporal imaging captured at intervals of the order of 10 s between frames, revealed structural evidence of polymerizing hyaluronan chains with increasing length with time. The imaging approach supported determination of hyaluronan polymerization rate at the individual enzyme level, and also determination of rate at various extent of polymerization. Such an approach indicates the high level of detail of information that can be extracted from such imaging studies.

**FIGURE 4:**
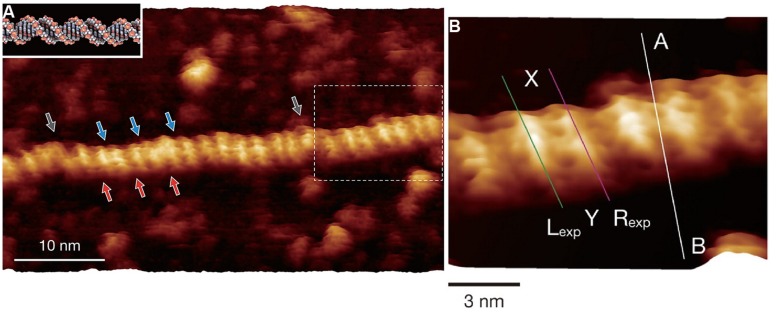
**Force modulation AFM topographs of plasmid pUC18 DNA (2686 basepairs) in aqueous solution**. The aqueous solution contained Ni^2+^ to stabilize the interaction between DNA and mica. **(A)** High resolution force modulation AFM topograph of a section of the duplex DNA with major (red arrows) and minor (blue arrows) grooves identified along the B-DNA double-helical structure. The white dotted area of the topograph in **(A)** are shown at higher magnification in **(B)**. **(B)** Height variations along the **(A–B)** cross-sectional line are shown in the original publication. Height profiles along the L_exp_ and R_exp_ lines are compared with simulated data in the original publication. Reprinted with permission from Ido et al. (2013). Copyright (2013) American Chemical Society.

While there are examples of application of recent development of high speed AFM for understanding biological functions relevant for EPS, the utilization of the more recently developed higher resolution imaging modalities to EPS is so far essentially lacking. The resolution in the obtained AFM topographs depend on a number of factors such as radius of curvature of the scanning tip, force control during the data acquisition, mechanical properties of the specimen. Development of higher resolution strategies within the scanning probe platform relevant for imaging of EPS include, e.g., the use of alternative feedback signals like the maximum force (PeakforceTM^®^, and others), exploitation of cantilever drive oscillations with more than one frequency (Garcia and Herruzo, 2012; Herruzo et al., 2013) and multimodal intermodulation AFM (Borysov et al., 2013), and ultra-low noise with improved force sensitivity. It should, however, be noted that not all imaging modalities are yet implemented for operation in liquid environment. Although not being an example of observation of an EPS, the recent report on imaging localized protrusion along the DNA double helical structure consistent with localization of phosphate groups is intriguing (Ido et al., 2013; **Figure 4**), and could also stimulate the use of such high resolution AFM techniques to EPS. In their study, Yamada and coworkers, deposited their nucleotide samples on freshly cleaved muscovite mica and rinsed with a solution containing Ni^2+^, one of the divalent ions that has been reported to substitute with the K^+^ site in the surface of the muscovite mineral, and thereby mediate a cationic bridge interaction to polyanions like DNA when imaged in aqueous solution (Hansma and Laney, 1996). The AFM employed was modified from a commercial one focusing on improving the force detection and also operating at minute oscillation amplitudes (0.4–0.5 nm). The obtained AFM topographs (**Figure 4**) resolve minor and major grooves of the dsDNA similar similar to other reports (Leung et al., 2012; Pyne et al., 2014), but even more impressing is the additional identification of the protrusions identified to arise from the phosphate groups. Application of such high resolution force modulation AFM to EPS could potentially contribute to resolving structural detail beyond that currently available. In addition to the general quest for higher resolution also relevant for EPS, particular issues related to, e.g., direct localization of acetyl groups along microbial alginate (Davidson et al., 1977) and gellan gum (Giavasis et al., 2000) could add to current approaches elucidating localization of substituents.

Scanning probe imaging tools as the AFM can also be operated to obtain information on the mechanical properties of a specimen, either as topographically resolved information of the biological entity of interest like a baceterial polysaccharide, or within constructs designed to probe molecular conformation. Pulling experiments on isolated macromolecules have developed to a large field providing novel, fundamental information on structure of macromolecules (Noy, 2008; Bizzarri and Cannistraro, 2010; Noy and Friddle, 2013; Eghiaian et al., 2014). The forces applied and the molecular extensions range typically from tens to hundreds of pico-newtons (pN) and a few to tens of nanometers, respectively, in these approaches. For polysaccharides, the force-extension relationships are conventionally obtained by stretching chains adsorbed between a substrate and an AFM canitelever (Rief et al., 1997; Marszalek et al., 1998; Sletmoen et al., 2010; Marszalek and Dufrene, 2012). Probing the mechanical properties of EPS are expected to provide data related to their mechanical and viscoelastic properties extending also to elastic responses associated with deformation of individual sugar residues. Although of interest and relevance for the functional properties of EPS, details within this topic is considered beyond the scope of the present review. The reason for this is that possible fingerprint signatures arising from stretching individual EPS have not yet been implemented as a possible contrast signature in AFM based imaging modalities.

While the above examples on the application of scanning probe based imaging modalities was mainly highlighting examples where the EPS is either isolated from its parent organism, or studied in combination with other macromolecules, there is also increasing applications of this toolbox in conditions closer to the natural habitat.

Dufrêne (2008) has recently summarized application of AFM based strategies to obtain image based information related to structure, chemical composition, nature of interaction forces, and specific molecular recognition as part of analysis of microbes at the nanoscale. Various examples of the application of more recent application of such strategies have been summarized (Sletmoen et al., 2010). Combined, the examples include studies of effect of inhibition of the biosynthetisis of arabinans (a major cell wall component), differences in EPS topology in bacterial mutants, and effects of EPS in cellular adhesion. Despite that individual EPS chains are not clearly resolved in such studies consequences of their changes are clearly emerging. The presence and overall outline of the polysaccharide capsules of the bacteria Zunongwangia profunda SM-A87 was recently characterized by tapping mode AFM (Su et al., 2012). In this report, the AFM clearly revealed the overall outline of the EPS containing capsules, the fibrils, but not individual, dispersed polysaccharide. The authors compared the recently implemented ScanAsyst mode from one commercial supplier and compared physical paramenters obtained by this mode to tapping mode. The recently reported mechanical and adhesive properties mediated by capsular EPS of *Lactobacillus johnsonii* FI9785 (Dertli et al., 2013) and changes in the EPS functionalities assocated with genetically modified variants affecting the biosynthesis of one of the EPS’s, illustrate the applicability of the imaging technology also for such studies. Specific adhesion studies were realized using a lectin from *Pseudomonas aeruginosa* (PA1) covalent attached to the AFM probes using a flexible linker, and used for adhesion mapping of the native and genetically modified bacteria. Reduced adhesion in the mutated bacteria probed under aqueous solution showed the reduction of D-Gal in the strains genetically modified to reduce the expression of one of the two EPS. Such a study indicate the capability of the scanning probe tools to provide structural related information of capsular polysaccharides based also on the specific compostion. More recently, detailed surface characterization of the human pathogen *Sterotcoccus agalectiae* using AFM combined with electron microscopy has been reported (Beaussart et al., 2014).

While the force based scanning probe modalities outlined above provide important structural and functional information, they are not directly providing information on chemical compostion. There are additional imaging tools sensitive to chemical information that is of potential interest for application to EPS. TERS has attracted interest for application in life sciences because it potentially can be used to obtain Raman spectra localized to certain domains. Application of TERS to EPS appears so far to be limited. Although not being a microbial alginate, we summarize some features of alginate fibers obtained by application of TERS (Schmid et al., 2008). Toward this end, an aliquot of a 2% alginate solution was drop-coated on cleaned glass surface and allowed to dry and characterized by TERS in a customized scanning probe – Raman spectrometer set-up. Commercial contact mode AFM tips coated with AlF_3_ (30 nm) and subsequently with Ag (30 nm) were prepared in a vapor coating system and used for the TERS characterization. The observed most prominent Raman bands determined by TERS were correlated with that of literature data and strategies for specta correction were suggested. The more recent overview of application of TERS for characterization of biological molecules provides guidelines considered relevant also for the EPS (Blum et al., 2012).

## Optical Based Imaging Modalities and their Applications to Microbial EPSs

While scanning probe techniques provide resolution which is far superior to optical techniques, they are limited to interrogating only the surface properties of the sample. Optical techniques have the advantage of being able to volumetric images without sectioning and also necessitates less sample preparation implying that samples can be imaged in their native environment. Over the past century it was believed that the resolution limit of far-field optical microscopy was fundamentally limited by the diffraction limit which according to Abbe’s theory limits the minimum focal area (Airy disk) to about half of the excitation wavelength. This paradigm has now been challenged. Over the last decade it has become clear that the diffraction limit is not an insurmountable barrier to resolution. Several concepts have evolved which provide resolution well beyond the resolution provided by conventional confocal microscopy.

These superresolution (or nanoscopy) techniques can be roughly divided into three categories. *Single molecule localization* techniques use the fact that the signal from a single molecule can be localized with a precision much greater than the size of the Airy disk. By using different concepts to sequentially turn fluorophores on and off, a superresolution image can be generated by combining multiple (often thousands) frames. Fluorophores can be turned on or off either stochastically (stochastical optical reconstruction microscopy –STORM Rust et al., 2006) or actively using light (Photoactivated light microscopy Betzig et al., 2006). The *RESOLFT* (Reversible Saturable OpticaL Fluorescence Transision) concept (Hofmann et al., 2005) is based on using a doughnut shaped beam which can turn off molecules reversibly [e.g., by stimulated emission (STED; Hell and Wichmann, 1994) or through ground-state depletion (GSD; Hell and Kroug, 1995)], reducing the volume of molecules which can still fluoresce. Structured illumination microscopy (SIM) illuminates the sample with structured light at different angles and is able to extract superresolution information through subsequent computational techniques (Gustafsson, 2005). These superresolution techniques have in the last few years provided novel results in many areas of microbiology. Especially new information about organization of proteins, enzymes, and receptors have been gained through the use of these superresolution techniques (Chojnacki et al., 2012). In the field of microbial EPS the techniques show great promise but have not yet been widely applied. Part of the reason might be that the scientific questions in the field are focused at structural information which is still beyond the resolution of optical superresolution techniques, requiring, e.g., electron microscopy or AFM.

**FIGURE 5:**
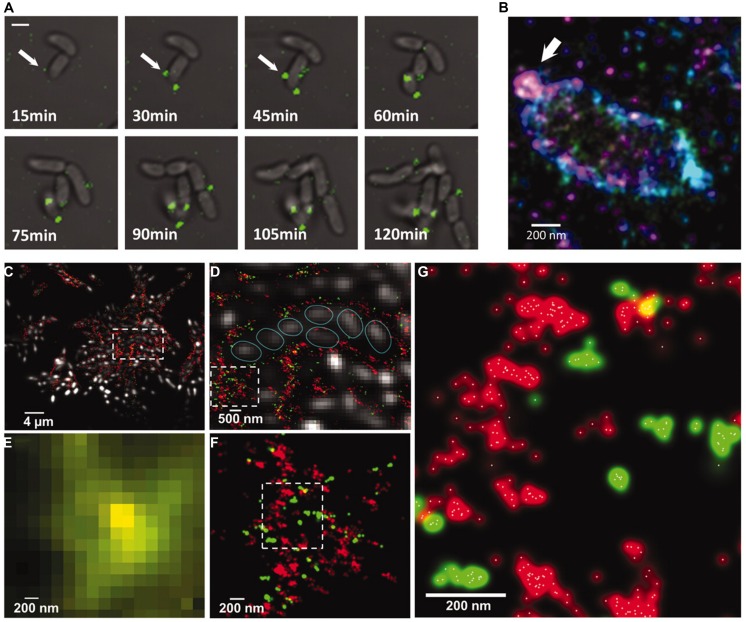
**(A)** Sectretion of *Vibrio* polysaccharide (VPS) stained with Cy3 attached to wheat germ agglutinin (green). **(B)** 3D STORM superresolution image of a single cell showing sectretion of VPS around a single cell. The color represents height in the *z*-direction. **(C–F)** Multicolor microscopy images of VPS (red) and RbmC (green). **(C,D,F)** are STORM superresolution images while **(E)** is a conventional confocal of the same region as **(F)**, illustrating the increased understanding of the organization of VPS and RbmC in superresolution microscopy. **(G)** STORM image showing the individual localization points as white dots. Reprinted with permission from Berk et al. (2012). Copyright AAAS.

Another issue is that most optical techniques require labeling with fluorophores and the availability of these are much larger for proteins than carbohydrates. However, in the study of the dynamics and interactions between the EPS, linking proteins and enzymes, the techniques seem to hold much promise. This was nicely illustrated in the study of the EPS and linking proteins in *Vibrio cholera* (Berk et al., 2011, 2012; **Figure 5**). STORM revealed that polysaccharides were extruded as distinct spheroids from the cells. It was shown that in the extracellular matrix (ECM), the polysaccharides and crosslinking polymers appeared in distinct clusters which could be a reason for the rapid repair of the ECM after rupture. There are some studies of polysaccharides from non-microbial sources, e.g., plant cell walls using superresolution microscopy which demonstrates the application of the technique which could be extended to microbial sources of ECM (Moran-Mirabal, 2013).

While optical microscopy based on fluorescent labels is extensively used and can provide images with high specificity for a given molecule, the necessity of labeling will always to some degree perturb the biological system under investigation. In addition, since an attached label, rather than the molecule of interest, is probed, it is not possible to extract further molecular information by analyzing the optical properties of the molecule. Label-free techniques, which directly probe the properties of the molecule of interest, show great potential for extracting subresolution information. Confocal Raman microscopy probes the vibrational levels of the molecules. This can provide detailed molecular information but is hampered by weak signals and long acquisition times. This limitation can to some degree be overcome by local enhancement effects as in the above mentioned TERS technique. Another class of label-free techniques is based on the non-linear optical properties of the sample. Non-linear optical microscopy (NLOM) has over the last decades evolved to become an important tool in biological research. Two-photon excited fluorescence (TPEF) was developed to overcome some of the limitations in conventional confocal microscopy (Helmchen and Denk, 2005), that is limited by sample penetration and out-of-focus photobleaching. NLOM is inherently confocal so that energy is only absorbed in the focal volume, reducing out of focus bleaching. Imaging down to 1 mm has been reported (Kobat et al., 2009). This feature could provide novel insight into the volumetric organization of EPS rather than only the surface, as is the case with, e.g., AFM.

In recent years several other non-linear interaction mechanismshave gained interest in biological research. The primary driver for these methods is that they provide label-free imaging of various molecules with relatively high specificity. They are therefore highly suitable for *in vivo* and even *in situ* imaging. In terms of studying EPS, the most important aspect of these techniques is the ability to image dynamic processes at very short timescales, without the necessity of introducing exogenous labels which might disturb the biological system, and where labeling efficiency is an unavoidable issue. Examples of mechanisms which have been studied dynamically are: secretion of cellulose from bacteria (Brackmann et al., 2010), hydration of starch (Slepkov et al., 2010) and digestion by cellulose (Brown et al., 2003).

Second harmonic generation (SHG) is a non-linear process where two photons combine to a single photon with twice the energy (half the wavelength) through the interaction with a molecule (Campagnola and Loew, 2003). The specificity of SHG arises from the fact that only molecules which are non-centrosymmetric and are ordered on the scale of the focal volume can generate SHG. Collagen is the most widely studied molecule with SHG owing to its non-centrosymmteric helical structure and that several collagen types order into fibrils (Lilledahl et al., 2007; Olderoy et al., 2014). Other molecules which can generate SHG are tubulin (Mohler et al., 2003; Stoothoff et al., 2008) and myosin (Plotnikov et al., 2006). Cellulose has also been shown to give a SHG signal. As SHG is very sensitive to the length scale on which the molecules are ordered it can provide the degree of crystalline ordering in the extracellular microbial cellulose. The extracellular cellulose of *Acetobacter* and *Valonia* was also studied by SHG (Brown et al., 2003; Nadiarnykh et al., 2007). By studying the polarization resolved SHG signal as well as the forward/backward scattering pattern of the SHG signal, the orientation and degree of ordering of the fibers in the lamella were analyzed.

Coherent anti-Stokes Raman scattering (CARS) is a technique which uses two femtosecond lasers which interacts with vibrational modes of the molecules, similarly to Raman microscopy (Zumbusch et al., 1999). The advantage of CARS is that for a moderately strong vibrational mode the signal is much stronger such that imaging of live samples at high framerates is possible (Evans et al., 2005). In addition, CARS also has the same 3D sectioning capability as other NLOM methods. CARS is, just like SHG, a coherent effect so that the total signal is highly dependent on the ordering of the molecules in the focal volume, thereby providing structural information below the resolution limit. Cellulose synthesis by *Acetobacter xylinum* was imaged using a combination of SHG and CARS (Brackmann et al., 2010). The SHG signal was used to image the synthesis of the cellulose while the bacteria where imaged using the (unspecific) CH_2_ vibration detected by CARS. Development of the cellulose network was imaged non-invasively and label-free over 7 days. SHG was also used to study the interaction of human cells (Osteoprogenitor cells and smooth muscle cells) with the bacterial cellulose matrix as a potential scaffold in tissue engineering applications (Brackmann et al., 2011, 2012). CARS has only been used to image cellulose from plants and not microbial cellulose, but there is no reason why it should not be possible with microbial cellulose as well. It was shown that using polarization resolved CARS, distinct domains with different degrees of crystallinity was observed along the cellulose fibrils (Zimmerley et al., 2010).

To summarize, optical techniques can provide information from dynamic, living systems in 3D. Labeling techniques makes it possible to track multiple molecules simultaneously to study how the EPS interacts with other molecular species in the ECM. Non-linear, label-free, techniques provide an even less invasive imaging modality with the possibility of extracting sub-resolution structural information from the data.

## Aspects of Preparation of EPS Samples for Imaging

Above, major advancement in the development of various imaging modalities, either scanning, probe or optically based, and their application to EPS or samples related to EPS, have been highlighted. For all these application, there is in most cases a need for the preparation of the sample in some way. The need for sample preparation depends on the operation environment of the imaging tool and the contrast mechanism exploited. For instance, in the case of AFM, the sample needs to be immobilized to withstand the actual forces imposed during the image acquisition. Although there is increasing control of the force, including reducing the magnitude of the force excerted by the tip on the specimen, there is still a need for adherence of the biological specimen to a sample surface, while being able to keep the sample in a hydrated environment to preserve a condition mimicking the natural state. The use of ionic bridges mediated by divalent ions, e.g., Ni^2+^, between mica and biomolecules (DNA) have been reported (Hansma and Laney, 1996; Raigoza et al., 2013) to provide sufficient strength in the non-covalent immobilization to allow scanning probe based imaging. Although there are instrumental developments that to a larger extent is based on inherent properties of biological specimens, and thus termed label-free, these approaches are not yet sufficiently established to replace all optically based imaging modalities. The use of fluorescence labels is therefore expected to be instrumental also in the further advancement for the characterization of EPS by optical imaging methods. In this context, the tools available for fluorescence labeling of EPS are considered not to be as versatile as the current state of the art for application in other families of biomacromolecules, and progress in imaging of EPS is also expected to benefit from advancement of novel labeling schemes.

## Conclusion

Advancement of instrumentation and application of novel imaging strategies for determination of properties of extracellular polysaccharides has provided novel insight into this group of materials. Important examples include the possibility to capture information related to the dynamics in the processing of this group of biopolymers, as highlighted by the application of high speed AFM to capture relocation of a collection of cellobiohydrolase on their crystalline substrate while catalyzing the bioconversion (Igarashi et al., 2011). Despite the significant advancement in the AFM image capturing speed realized over the past decade, capturing key features of the dynamics associated with biomolecule movements are still not achievable by high resolution imaging (Raigoza et al., 2013). The situation for the high resolution AFM techniques is that the intriguing resolution reported for, e.g., B-DNA helix structures, should inspire migration of this technique to a number of interesting research questions within EPS. Added to this, is the advancement both in improved resolution beyond the diffraction limited barrier and label free tools, that is finding its way also into the EPS field. Moreover, there is also advancement in the imaging field bolstered by the capacity to observe identical locations of the sample using a combination of imaging tools, either as combined instruments, including spectroscopy (Moreno Flores and Toca-Herrera, 2009; Lucas and Riedo, 2012), or by the application other strategies. Thus, we conclude that the ultramicroscopy toolbox available for EPS are more richly developed than ever, and we expect further advancement in this field, with respect to improved dynamics, more precise structural, and localized chemical information potentially being unraveled to underpin improved understanding of the structure–function relationships.

## Conflict of Interest Statement

The authors declare that the research was conducted in the absence of any commercial or financial relationships that could be construed as a potential conflict of interest.
